# Long-term efficacy of triple semicircular canal plugging in the treatment of patients with ipsilateral delayed endolymphatic hydrops

**DOI:** 10.1038/s41598-021-82683-6

**Published:** 2021-02-04

**Authors:** Daogong Zhang, Yafeng Lv, Xiaofei Li, Yawei Li, Yongdong Song, Zhaomin Fan, Haibo Wang

**Affiliations:** Department of Otolaryngology-Head and Neck Surgery, Shandong Provincial ENT Hospital, Cheeloo College of Medicine, Shandong University, Jinan, 250022 People’s Republic of China

**Keywords:** Diseases, Signs and symptoms

## Abstract

This study aims to explore the long-term efficacy of triple semicircular canal plugging (TSCP) in the treatment of intractable ipsilateral delayed endolymphatic hydrops (DEH), so as to provide an alternative therapy for this disease. Forty-eight patients diagnosed with ipsilateral DEH referred to vertigo clinic of our hospital between Dec. 2010 and Dec. 2017, were included in this study for retrospective analysis. All patients were followed up for 2 years. Vertigo control and auditory functions were measured and analyzed. Pure tone audiometry, caloric test, and vestibular evoked myogenic potential (VEMP) were performed in two-year follow-up. Forty-five patients who accepted intratympanic gentamicin (26.7 mg/mL) twice given one week apart were selected as a control group. The total control rate of vertigo in TSCP group was 97.9% (47/48) in the two-year follow-up, with complete control rate of 83.3% (40/48) and substantial control rate of 14.6% (7/48). The rate of hearing loss was 22.9% (11/48). The total control rate of vertigo in intratympanic gentamicin group was 80.0% (36/45), with complete control rate of 57.8% (26/45) and substantial control rate of 22.2% (10/45), and the rate of hearing loss was 20.0% (9/45). The vertigo control rate of TSCP was significantly higher than that of intratympanic gentamicin (*χ*^2^ = 6.01, *p* < 0.05). There was no significant difference of hearing loss rate between two groups. (*χ*^2^ = 0.12, *p* > 0.05)**.** TSCP, which can reduce vertiginous symptoms in patients with intractable ipsilateral DEH, represents an effective therapy for this disorder.

## Introduction

DEH is characterized by vertigo like Meniere’s type occurring in patients with pre-existing ear pathology, including serious unilateral hearing loss caused by trauma, infection, or other unknown events in childhood^1,2^. DEH can be classified as three types including bilateral, ipsilateral or contralateral. The histopathologic changes of deaf ears were found to be in keeping with viral labyrinthitis, whereas the changes in hearing ears were consistent with those seen in patients with Meniere's disease, as shown by pathologic analysis of temporal bones of two contralateral DEH patients^3^.

The first-line treatment of ipsilateral DEH is medical therapy, with surgery indicated when medical treatment fails to control vertigo. Labyrinthectomy is found to be curative in patients with ipsilateral DEH, which however would result in total deafness. Intratympanic gentamicin, a minimally invasive outpatient procedure for refractory Ménière’s disease, also is generally used for DEH patients. But the drawback of gentamicin therapy is obvious as that the dose of gentamicin, the frequency of injections, the number of doses to give, and clinical endpoints for therapy are still not well established.

Semicircular canal occlusion has been shown to be effective in the treatment of patients with intractable benign paroxysmal positional vertigo and in those with intractable peripheral vertigo^4–6^. In our previous study, we reported the results of MD patients who accepted triple semicircular canal plugging (TSCP). The vertigo control rate was excellent as about 98% in two-year follow-up^7–9^. This study assessed the long-term efficacy of TSCP in the treatment of DEH and compared the effects between TSCP and intratympanic gentamicin.

## Results

In this work, the demographic information for the TSCP and intratympanic gentamicin groups was presented in Table 1. Briefly, the age, gender, and duration of disease of patients with TSCP had no significant differences with that of patients with intratympanic gentamicin.

**Table 1:** The demographic information of patients with TSCP and intratympanic gentamicin patients.

Group	Gender		Average age	Average duration (moths)	Hearing thresholds (dB)
	Male	Female			
TSCP	22	26	53.0	71.8	63.7
Intratympanic gentamicin	22	23	53.5	59.7	63.2
Statistic value	χ^2^ = 0.087		t = 0.248	z = 0.286	t = 0.883
*P* Value	> 0.05		> 0.05	> 0.05	> 0.05

The total control rate of vertigo in TSCP group was 97.9% (47/48) in the two-year follow-up, with complete control rate of 83.3% (40/48) and substantial control rate of 14.6% (7/48). (Table 2) The rate of hearing loss was 22.9% (11/48). (Table 3).

**Table 2:** The control rate of vertigo in TSCP and intratympanic gentamicin patients.

Group	Total cases	Vertigo control rate	χ^2^ Value	*P* Value
TSCP	48	97.9%	6.01	*P* < 0.05
Intratympanic gentamicin	45	80.0%

**Table 3:** The hearing loss rate in TSCP and intratympanic gentamicin patients.

Groups	Total cases	Hearing loss rate	χ^2^ Value	*P* Value
TSCP	48	22.9%	0.12	*P* > 0.05
Intratympanic gentamicin	45	20.0%		

Post-operatively, all 48 patients experienced temporary vertigo and balance disorders. Vertigo disappeared in all patients within 5 days, while balance disorders disappeared in 37 patients within 1–2 weeks after surgery and in the other 11 patients within 2 months. The average recovery time was 15.2 days.

Prior to surgery, 30 patients (62.5%) had abnormal results on caloric tests, with a poor response on the affected side. Twenty-nine patients (60.4%) had abnormal cVEMPs, with a decreased amplitude in the affected ear. Caloric tests showed canal paresis on the operated side of all 48 patients 24 months after treatment. Moreover, 31 patients (64.6%) had abnormal postoperative cVEMPs, which had not significant difference compared with the results detected preoperatively (*χ*^2^ = 0.18, *p* > 0.05). (Table 4). Nineteen patients with a normal preoperative cVEMP continued to have a normal postoperative cVEMP, whereas 2 patients with a normal preoperative cVEMP had an abnormal postoperative cVEMP. Twenty patients accepted oVEMP test. Ten patients (50.0%) were abnormal in oVEMP with a decreased amplitude in affect ear. oVEMP presented 55.5% (11/20) abnormally postoperatively, which had not significant difference compared with the results detected preoperatively (*χ*^2^ = 0.10, *p* > 0.05). (Table 5) Nine patients with a normal preoperative oVEMP continued to have a normal postoperative oVEMP, whereas only 1 patient with a normal preoperative oVEMP had an abnormal postoperative oVEMP. None of the 48 patients experienced facial paralysis, cerebrospinal fluid leakage, or other complications.

**Table 4:** Abnormal rate of cVEMP pre- and post-operatively in TSCP and intratympanic gentamicin patients.

Group	Preoperative	Postoperative	χ^2^ value	*P* value
TSCP	60.4%(29/48)	64.6%(31/48)	0.18	*P* > 0.05
Intratympanic gentamicin	53.3%(24/45)	88.9%(40/45)	13.85	*P* < 0.05

**Table 5:** Abnormal rate of oVEMP pre- and post-operatively in TSCP and intratympanic gentamicin patients.

Groups	Preoperative	Postoperative	χ^2^ value	p value
TSCP	50.0%(10/20)	55.0%(11/20)	0.10	p > 0.05
Intratympanic gentamicin	47.6%(10/21)	85.7%(18/21)	6.86	p < 0.05

**Figure 1:**
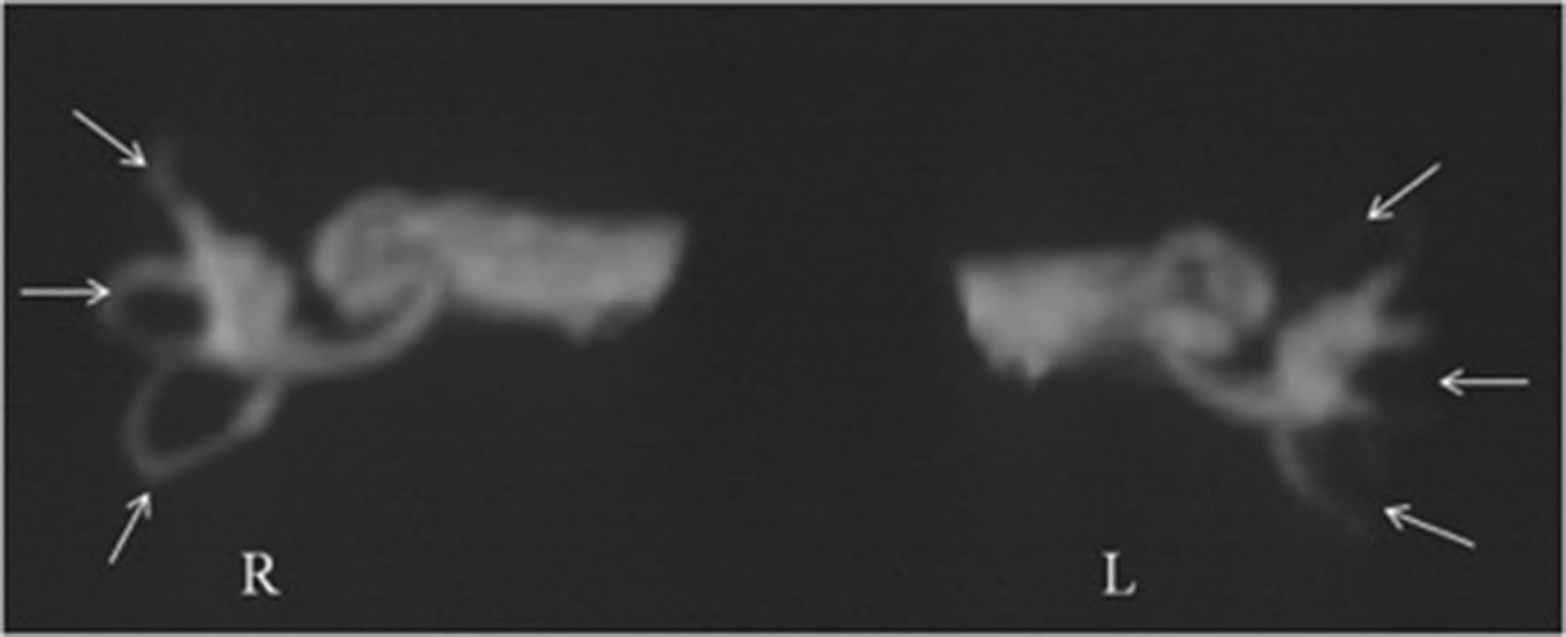
Magnetic resonance imaging of the inner ear fluid 2 years after TSCP of a 69-year-old patient with DEH of the left ear. Endolymph fluid in the position of plugging of the three semicircular canals of the left (pathological) ear contained no water (arrows), whereas endolymph fluid of the right (healthy) ear was normal. *SSC* superior semicircular canal; *HSC* horizontal semicircular canal; *PSC* posterior semicircular canal; *R* right ear; *L* left ear.

Magnetic resonance hydrography of the inner ear 2 years after TSCP showed that endolymph fluid in the position of plugging contained no water (Fig. 1). Magnetic resonance hydrography using heavily T2 weighted imaging showed that endolymph fluid of the inner ear had a long T2 relaxation time. Magnetic resonance was performed in all the patients 2 years after TSCP, and the absence of water in the position of plugging was confirmed in all of them.

The total control rate of vertigo in chemical labyrinthectomy group was 80.0% (36/45), with complete control rate of 57.8% (26/45) and substantial control rate of 22.2% (10/45). The rate of hearing loss was 20.0% (9/45).

The vertigo control rate of TSCP was significantly higher than that of chemical labyrinthectomy (*χ*^2^ = 6.01, *p* < 0.05). (Table 2) There was no significant difference of hearing loss rate between two groups. (*χ*^2^ = 0.12, *p* > 0.05)(Table 3).

In intratympanic gentamicin group, 27 patients (60.0%) had abnormal caloric test presenting a poor response in affected side prior to operation. Twenty-four months after treatment, canal paresis was found in the operation side of 37 patients (82.2%) by means of caloric test. 24 patients (53.3%) were abnormal in cVEMP with a decreased amplitude in affect ear. cVEMP presented 88.9% (40/45) abnormally postoperatively, which had significant difference compared with the results detected preoperatively (*χ*^2^ = 13.85, *p* < 0.05 ) (Table 4). Twenty-one patients accepted oVEMP test. Ten patients (47.6%) were abnormal in oVEMP with a decreased amplitude in affect ear. oVEMP presented 85.7% (18/21) abnormally postoperatively, which had significant difference compared with the results detected preoperatively (*χ*^2^ = 6.86, *p* < 0.05) (Table 5).

The average recovery time of imbalance in intratympanic gentamicin group was 21.8 days, which was much longer than that of TSCP group. (t = 5.57, *p* < 0.05) (Table 6).

**Table 6:** The average recovery time of imbalance in TSCP and intratympanic gentamicin patients.

Groups	Average recovery time (days)	Statistic value	p Value
TSCP	15.2	5.57	< 0.05
Intratympanic gentamicin	21.8		

## Discussion

Although there is currently no cure for DEH, most patients with this disorder are helped by medical treatment. When conservative treatment fails to control vertigo successfully with ipsilateral DEH, chemical labyrinthectomy or labyrinthectomy are taken into account.

Semicircular canal plugging has been a new method to treat MD in recent years^5,6^. TSCP had no effect on cochlear function in animal models of EH^11,12^. Lateral canal plugging resulted with 75% vertigo control in 28 patients with MD^5^. Use of TSCP to treat 3 MD patients who previously underwent unsuccessful endolymphatic sac surgery resulted in complete or substantial vertigo control in these patients^6^. We previously used TSCP to treat intractable MD patients and obtained excellent results in controlling vertigo during 2 years follow up^7–9^. To date, however, TSCP has not been reported using in patients with DEH. We found that TSCP in 48 patients with DEH effectively controlled vertigo in 47 (97.9%) of these patients after 2 years. Caloric tests affirmed that TSCP induced canal paresis on the surgery side in all 48 patients, suggesting that this surgery was capable of controlling vertigo by removing functions of semicircular canals. Plugging of three semicircular canals may block endolymph fluid and greatly reduce its movement within canals, resulting in minimal stimulation of crista ampullaris during angular movement. None of the operated ears in the present study responded to caloric stimulation, indicating that fluid motion induced by caloric stimulation was interrupted. Magnetic resonance hydrography of the inner ear 2 years after TSCP affirmed that there was no water in the canals at the position of plugging, further confirming the above supposition.

We also discovered that the abnormal rate of VEMP tests did not differ significantly between before and two years after TSCP, indicating that TSCP did not damage the function of otolith organs. Therefore, vestibular compensation could occur more rapidly with TSCP than with labyrinthectomy or vestibular neurectomy. Previous research showed that only a half of labyrinthectomy patients could return to work even though most of them are relieved of vertigo^11^. Another study showed that vestibular neurectomy resulted in a longer delay before returning to work^12^. Most patients showed disappearance of imbalance within 1–2 weeks after surgery. Similarly, an animal study of cats showed that recovery was discovered to be more quickly underwent TSCP than that underwent labyrinthectomy^13^. In addition, TSCP had no effect on the resting discharge of the hair cells of vestibular end organs, which was good to rehabilitation of imbalance^14^.

Unlike labyrinthectomy or chemical labyrinthectomy, TSCP interrupts endolymphatic flow in the canals, but sensorineural cells are preserved, being still capable of suffering for mechanical or chemical injuries. In the same way, preservation of the otolith function is frequently claimed to cause ablative techniques failure and several studies have shown that abolition of otolith responses predicts better vertigo control^15–17^. In TSCP, long term good results in vertigo control are linked to preservation of otolith function. We think that it might be due to the fact that rotatory vertigo was induced most by abnormal responses of semi-circular canals but not the function of otolith. Additionally, besides interrupting endolymphatic flow in the canals, some other mechanisms, which we do not know yet, might also exist in controlling vertigo by TSCP. Intratympanic gentamicin reduces vertigo, but inevitably impairs the function of the hair cells of vestibular end organs^18^. In this study, the abnormal rate of cVEMP and oVEMP postoperatively of chemical labrinthectomy group was much higher than that of preoperatively. The toxicity to vestibular hair cells of gentamicin has been extensively investigated. Gentamicin gathers mainly in vestibular hair cells of type 1 and leads to atrophy of these hair cells as well as the neuroepithelium^19^. TSCP had no damage to these cells of vestibular end organs and cochlea by comparison with intratympanic gentamicin. The outcomes of VEMP tests did not differ significantly between before and two years after TSCP, indicating that TSCP did not damage the otolith organs, and so we speculated that the TSCP controlled vertigo just through interrupting the motion of endolymph within three semicircular canals. Therefore, our data indicated that TSCP was less destructive theoretically by comparison with chemical labyrinthectomy. Iwasa et al.^20^ reported that in 19 cases of DEH, 6 patients were diagnosed with bilateral DEH (31.6%). So we should pay careful attention to this bilateral pathology and give priority to TSCP when compared with intratympanic gentamicin.

In this study, we compared the vertigo control rate of TSCP and intratympanic gentamicin. Our data showed that the vertigo control rate of TSCP was much higher than that of intratympanic gentamicin group, suggesting that TSCP has advantages in vertigo control compared with intratympanic gentamincin. The reason might be that TSCP could block the movement of the endolymph semicircular canals, while the effect of intratympanic gentamicin is easy be affected with many factors such as permeability of round window membrane, function of eustachian tube, etc. Yoshioka M found that round window permeability was absent in 5% of ears, and 13% of ears had poor round window permeability^21^. Another explanation might be that a profound vestibular deafferentation is needed to efficiently control vertigo in disable MD patients^22^. Intratympanic gentamincin could not guarantee to obtain profound vestibular deafferentation.

This study found that the hearing loss rate was either about 20% either in TSCP group or in intratympanic gentamicin group. Thus we suggested that TSCP and intratympanic gentamicin should be used in those patients with MD who had unserviceable hearing. Flanagan et al. reported 81.3% of vertigo control and 21.4% of hearing loss after a single intratympanic injection of gentamicin, and another study reported 81% vertigo control and 12% of hearing loss after a maximum of 2 injections^23,24^. In this research, there was a 80.0% vertigo control and 20.0% hearing loss rate in intratympanic gentamicin group, which was in conformity to previous reports. There was a 22.9% hearing loss rate in TSCP group, with no significant difference compared to intratympanic gentamicin group. Although the cause of hearing loss in TSCP is not clear, perilymphorrhea or serious fibrous labyrinthitis might be the reason^25^.

As with the majority of studies, the design of the current study is subject to limitations. The main limitation of this work is the retrospective design of the study. The patients were not assigned to each group randomly. The second limitation is that the data were collected in a single institution. In the future, a prospective and multi-center study will be performed.

## Conclusion

This is the first report about using TSCP in treatment of DEH. Our study showed that TSCP could control vertigo effectively in patients with intractable DEH, indicating that TSCP is an efficacious surgical treatment for this disease. The vertigo control rate of TSCP was significantly higher than intratympanic gentamicin.

## Materials and methods

### Patients

This study enrolled 48 patients (22 men, 26 women; age range 33–71 years, mean 53.0 years) diagnosed with ipsilateral DEH according to the criterion put forward by Schuknecht^2^ and referred to the vertigo clinic of our hospital between December 2010 and December 2017. Of these 48 patients, all were clinically diagnosed with ipsilateral DEH. All patients received standard conservative treatment, consisting of betahistine 12 mg tid and hydrochlorothiazide 25 mg bid, for at least six months, but continued to experience vertigo, defined as at least two attacks per month in 3 months before surgery, with each attack lasting more than 20 min. All the patients had unserviceable hearing (PTA threshold > 60 dB or speech recognition rate < 50%). Cerebellopontine angle tumors, vestibular migraine and other central vertigo diseases were excluded. Forty-five patients (22 men, 23 women; age range 32–67 years, 53.5 years) who accepted intratympanic gentamicin (26.7 mg/mL) twice given one week apart during the same period were selected as a comparison group. All patients were clinically diagnosed with ipsilateral DEH. All patients received standard conservative treatment, consisting of betahistine 12 mg tid and hydrochlorothiazide 25 mg bid, for at least six months, but continued to experience vertigo. All the patients had unserviceable hearing (PTA threshold > 60 dB or speech recognition rate < 50%). The study was approved by the Ethics Committee of Shandong Provincial ENT Hospital, Cheeloo College of Medicine, Shandong University and all patients provided written informed consent. Our study involving humans is in compliance with the Declaration of Helsinki. We confirm that all methods were carried out in accordance with relevant guidelines and regulations.

### Surgical procedure of TSCP

TSCP was performed as described in our previous paper^7^. Briefly, surgery was performed under general anesthesia using a postauricular approach. Three bony semicircular canals were exposed through mastoidectomy. The bone was drilled up to the blue line in the central portion of the bony canal. A 2-mm segment of canal was skeletonized to create a fenestra, without opening either the endosteum or the membranous labyrinth. A plug of temporalis fascia was inserted through the fenestra to compress the endosteum and membranous labyrinth against the back bony wall. The fenestration was covered with bone wax to prevent perilymphorrhea, and the incision was closed^7^.

### Intratympanic gentamicin

Intratympanic gentamicin was performed as described in our previous paper^8^. Intratympanic injections were done in outpatient clinics. One phial of gentamicin sulfate (80 mg/2 ml) was first diluted with 1 ml of 5% NaHCO_3_. One milliliter of this solution, corresponding to 26.7 mg of gentamicin, was then injected in the postero-inferior portion of the tympanic membrane of the affected side upon topical anesthesia with a 2.5% lydocaine, under microscopic view and with the aid of a spinal needle. The patient was placed supine with the head rotated 45° contralaterally in respect to the ear to be treated and kept in this position for 30 min after the injection. Two injections were given, the second injection one week after the first^8^.

### Evaluation of vertigo

Evaluation of vertigo was carried out as described in our previous paper^7,8^. A definitive spell of vertigo lasting more than 20 min was regarded as a Meniere’s vertigo attack according to the AAO-HNS criteria issued in 1995^10^. Patients were instructed to record acute attacks of vertigo, coexisting symptoms (such as tinnitus, aural fullness, changes in hearing) and other characteristics included time of onset and duration in a paper-based diary for the full 24-month study duration. The frequency of definite vertigo attacks 6 months before treatment was compared with the frequency of attacks occurring between 18 and 24 months after treatment. The vertigo control after 2 years was established using the formula and criteria of the AAO-HNS and noted on a scale of A (complete control) to F (no control, secondary treatment). Patients with scale A (complete control) and B (substantial control) were defined as successful vertigo control^8^.

### Evaluation of hearing

Evaluation of hearing was carried out as described in our previous paper^7,8^. Hearing function was measured by a pure tone audiometer and was evaluated based on the four-tone average formulated by (a + b + c + d)/4 (a, b, c and d are hearing levels at 0.5 kHz,1 kHz, 2 kHz and 3 kHz, respectively) according to 1995 AAO-HNS criteria^10^. The worst hearing level during the 6 months of the surgical ear before treatment has been compared with the worst hearing level between 18 and 24 months after treatment. Changes greater than 10 dB in hearing level is “better” or “worse” and changes within 0–10 dB is “no change”^7,8^.

### Caloric test

Bithermal caloric tests were performed as described before^7^. Briefly, each ear was irrigated alternatively with a constant flow of air at 24 °C and 49 °C for 40 s. The response was recorded over 3 min, and a 7-min interval between each stimulus was respected to avoid cumulative effects. A video-based system was used (Ulmer VNG, v. 1.4; SYNAPSYS, Marseille, France) to acquire and analyze the eye response. The maximum slow-phase velocity (SPV) of nystagmus after each irrigation was calculated and, unilateral weakness (UW) was determined according to Jongkee’s formula. In our laboratory, the value of UW less than 20% was considered to be normal. The value of UW more than 90% was considered to be canal paresis^7^.

### VEMP test

VEMP tests were performed as described before^8^. From 2010 to 2013, only cVEMP was tested. From 2014 to 2017, both cVEMP and oVEMP were all tested.

From 2010 to 2013, cVEMPs were recorded using a Smart EP device (Intelligent Hearing Systems, USA). cVEMP test was carried out as described in our previous paper^8^. The electromyographic activity of the sternocleidomastoid muscle was recorded while patients were laying supine on a bed and asked to raise their head off of the bed in order to activate their neck flexors bilaterally and the saccular receptor were acoustically stimulated with air-conducted acoustic stimulation. The recording electrode was placed at the middle third of the muscle ipsilateral to the stimulated ear, the reference electrode was placed on the upper edge of the sternum and the ground electrode was placed on the sternocleidomastoid muscle contralateral to the stimulated side. Attention was paid to place bilateral electrodes on symmetrical site. The amplifier gain was set to 100,000, and signals and bandpass were filtered 10 to 3000 Hz. Short-tone bursts (100 dB nHL, 500 Hz, each, with 1 ms raise fall time and 5-ms plateau time) were delivered monaurally by TDH 49P earphones. The stimulation rate was 5 Hz; the analysis time was 60 ms. A total of 128 responses to stimuli were averaged, and measurements were repeated twice to check test wave reproducibility. A clearly defined biphasic response was recorded in the SCM ipsilateral to the side of cathode placement in all subjects. We refer to this as the p13/n23 response. We analyzed the amplitudes of the first positive–negative peak, p13-n23 and peak latencies of p13 and n23. The average of two runs was taken for the amplitudes and latencies. The amplitude ratio between the two ears over 1.61 was considered abnormal. The latency of p13 over 17.3 ms, while, n23 over 24.6 ms was considered abnormal^8^.

From 2014 to 2017, the recording system of VEMPs was using the Neuro-Audio auditory evoked potential equipment (Neurosoft LTD, Ivanov, Russia). VEMP tests was carried out as described in our previous paper^8^. The test was performed with the patients in the seated position. Tone burst stimuli were delivered via a standard insert earphone (ER-3A). Active recording electrodes with respect to the cVEMP examination were placed on the region of the upper third of the sternocleidomastoid muscle (SCM) on both sides. The reference electrodes were placed on the upper sternum. The ground electrode was on the nasion. The head was rotated towards the contralateral side of the stimulated ear to achieve tonic contraction of the SCM during recording. For the oVEMP, active recording electrodes were placed on the infra-orbital ridge 1 cm below the center of each lower eyelid and reference electrodes were positioned approximately 1 cm below them. The ground electrode was on the nasion. oVEMP were recorded with eyes open and maximal gaze upwards. The electrode impedance was maintained below 5kΩ and the stimulation rate was 5.1 Hz. The VEMPs were measured with 500 Hz tone burst and the initial intensity was 110 dB nHL. The intensity decreased from 110 dB nHL to the threshold in 5 dB steps. cVEMP superimposition number n is 60 times, oVEMP superimposition number 100 ≤ n ≤ 200, analysis time is 0–50 ms. Bandpass filtering of cVEMP is 30-2000 Hz and bandpass filtering of oVEMP is 1–1000 Hz. The latency of p1 over 17.3 ms, while, n1 over 24.6 ms of cVEMP was considered abnormal. The amplitude ratio over 30% was considered abnormal. The latency of n1 over 12.6 ms, while, p1 over 17.8 ms of oVEMP was considered abnormal. The amplitude ratio over 30% was considered abnormal^8^.

### Magnetic resonance hydrography of the labyrinth

Magnetic resonance imaging (MRI) scans were acquired using a 1.5 T MR unit (GE-signal, USA) as described before^7^. Briefly, three-dimensional fast imaging employing steady acquisition (3D-Fiesta) imaging was performed; the scan parameters for the 3D-Fiesta included a repetition time of 1.7 ms, an effective echo time of 5.0 ms, a matrix size of 320 × 384, and with 30 axial 0.8-mm thick slices to cover the labyrinth and internal auditory canal with a 180-mm square field of view. The number of excitations was two, and the scan time was 4 min. All MRI raw data were sent to a GE post-processing workstation and the maximum intensity projection (MIP) was used to reconstruct the structure of the labyrinth. MIP- reconstructed images were rotated once from 0 degrees in 15 degree steps to obtain multi-azimuth and multi-view images of the labyrinth and internal auditory canal^7^.

### Statistics

*χ*^2^ test, t-test and z-test were applied to compare demographic data of patients with TSCP and intratympanic gentamicin patients. *χ*^2^ test was applied to compare vertigo control and hearing loss rate among patients with TSCP and intratympanic gentamicin. *χ*^2^ test was applied to compare the abnormal rate of VEMP test before and after operation in TSCP and intratympanic gentamicin patients. t-test was applied to compare the average recovery time of imbalance after operation in TSCP and intratympanic gentamicin group. *p* < 0.05 was considered significant.
